# Effect of organic solvents on calcium minodronate crystal morphology in aqueous solution: an experimental and theoretical study†

**DOI:** 10.1039/d2ra07130d

**Published:** 2023-01-18

**Authors:** Chen Zhuang, Muyuan Chai, Yuhui Zhang, Xuetao Shi

**Affiliations:** a School of Materials Science and Engineering, South China University of Technology Guangzhou 510640 China; b School of Materials Science and Engineering, Xiamen University of Technology Xiamen 361024 China joanmolin@foxmail.com; c National Engineering Research Center for Tissue Restoration and Reconstruction, South China University of Technology Guangzhou 510006 China shxt@scut.edu.cn; d Key Laboratory of Biomedical Engineering of Guangdong Province, South China University of Technology Guangzhou 510006 China; e Key Laboratory of Biomedical Materials and Engineering of the Ministry of Education, South China University of Technology Guangzhou 510006 China; f Guangzhou Regenerative Medicine and Health Guangdong Laboratory 510005 Guangzhou China

## Abstract

The influence of nine organic solvents on the crystal morphology of calcium minodronate (Ca(Min)_2_) was investigated by experimental investigations and molecular simulations. Hirshfeld analysis was used to reveal the intermolecular interactions, and the modified attachment energy (AE) model was applied to constructing the Ca(Min)_2_–organic–water model in different organic–water solvents. The surface structure and the mass density profile were demonstrated and analyzed. The results showed that there were different adsorption conditions in different organic–water solvents. Furthermore, it was found that the (2 0 0)/(1 1 0) side ratio of Ca(Min)_2_ crystal had a linear relationship with the volume of organic solvent and had a certain correlation with some solvent properties. It is believed that the research developed in this work could have a promising application in prediction of Ca(Min)_2_ crystal morphology and could give guidance in the selection of organic solvents to control the desirable crystal morphology.

## Abstract 2

A modified AE model was applied to morphology prediction of Ca(Min)_2_ and relationships between morphology change and organic volumes were revealed.

## Graphical abstract

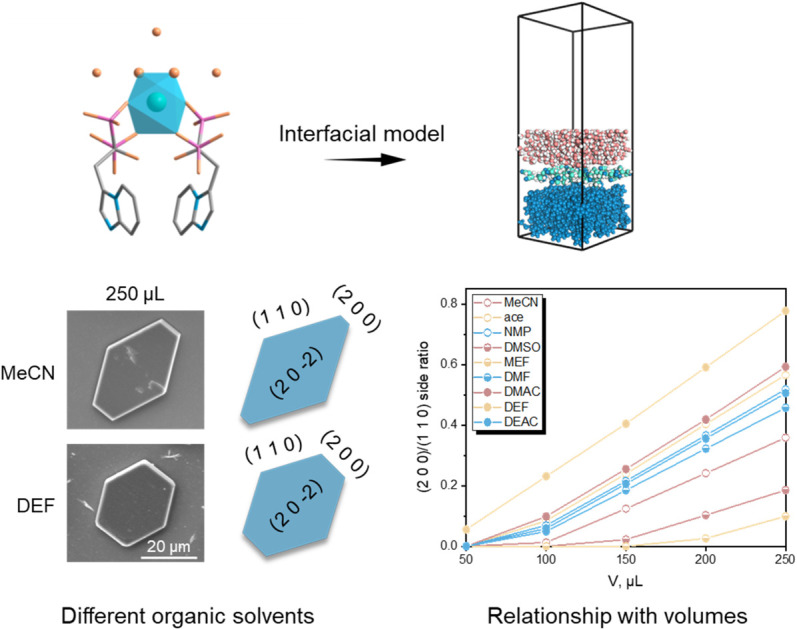

## 1. Introduction

Crystal morphology is the appearance of a crystalline material, which is one of the most important factors that can influence material properties, such as solubility, stability and bioavailability, as well as processing and packaging operations.^1–3^ Crystal morphology can be determined by various factors, including solvents,^4–8^ supersaturation^9,10^ and additives.^11–14^ Among them, solvents play a significant role in adjusting adsorption on certain crystal faces and should be preferentially taken into account. For example, Song *et al.*^15^ selected typical solvents based on their electronegativity to obtain different pronamide crystal shapes, and found that solvents with identified functional groups interacted with the crystal planes in different ways, which resulted in crystals with various aspect ratios. Cui *et al.*^16^ found that both the interface structure and the interaction strength for the crystal–solvent combination were important in the solvent effect on the sucralose crystal morphology, and the solvent–crystal interaction was a key factor, which was dominated by hydrogen bonds between hydrogen atoms of the crystal and oxygen atoms of the solvents. Despite all, to search for a desired crystal morphology of a material through experiments still remains expensive and time-consuming.

Recently, molecular simulation has provided new understanding of crystal morphology at the molecular level through computational models. Several theoretical models have been proposed to predict crystal morphology, such as non-mechanistic models including Bravais–Friedel–Donnay–Harker (BFDH) rule^17^ and attachment energy (AE) model,^18,19^ and mechanistic models including Monte Carlo method,^20,21^ interfacial structure analysis model,^22^ spiral growth model and 2D nucleation model.^9,23,24^ These theoretical models consider the influence on crystal morphology from the internal crystal structure to external environment, with increasing accuracy and complexity. Among these models, the AE model has generally become the most popular simulation method in crystal morphology prediction due to its inexpensive calculation and reliable accuracy.^25^ By considering the interaction energy between crystal faces and solvents, the modified AE model can be applied to crystallization in solutions. Li *et al.*^26^ studied the effects of solution conditions including solvents, temperature and supersaturation on β-HMX crystal morphology through molecular dynamics by the modified AE model, and the simulation results were well consistent with experiments. Liu *et al.*^27^ used the modified AE model to investigate the DNTF crystal morphologies in the solvents H_2_O/AcOH and H_2_O/EtOH, and further found that the (0 1 1) and (0 0 1) faces were morphologically important, which was in agreement with experimental results. Zhao *et al.*^28^ successfully extended the modified AE model to the morphology prediction of FOX-7 crystal in cyclohexanone, acetonitrile, H_2_O/DMF and H_2_O/DMSO, which was verified by the experiments. The results provided that H_2_O/DMF and H_2_O/DMSO were good solvents for recrystallization. Nevertheless, there is no reports about application of modified AE model on morphology prediction of metal organic coordination polymer.

During the synthesis of metal organic coordination polymer crystals, the addition of organic solvents can accelerate the reaction rate^29–31^ and change the crystal morphology.^32,33^ Polar aprotic organic solvents can significantly accelerate the reaction process of S_N_2 (Bimolecular nucleophilic substitution reaction), so they are commonly seen in the synthesis of metal bisphosphonates.^34–38^ However, the addition of such organic solvents have inevitable effect on the crystal morphology, thus changing the crystallization, reprocessing and bioavailability.^39–44^ Calcium bisphosphonates (CaBPs), one kind of novel coordination polymer of calcium and bisphosphonates, have revealed great potential in bone regeneration,^45,46^ anticancer drug^34^ and delivery system.^47^ Minodronate (Min) is believed as one of the most effective bisphosphonates (100–1000 times higher than pamidronate in terms of inhibiting bone resorption).^48,49^ As one type of CaBPs, calcium minodronate (Ca(Min)_2_) has been rarely investigated, especially in terms of crystal morphology. At present, there have been no experiment and simulation investigations into the influence of polar aprotic organic solvents on crystallization of Ca(Min)_2_. In this work, Ca(Min)_2_ was selected to study the effect of organic solvents on the crystal morphology. Molecular dynamic simulations were employed to reveal the morphology transformation in organic–water solvents. Experiments were conducted to demonstrate the correlation between the morphology change and the volume of organic solvent. The relationship between the morphology change and the organic solvent properties were also analyzed.

## 2. Experimental and simulation methods

### 2.1 Materials

Minodronate (Min, C_9_H_12_N_2_O_7_P_2_, CAS: 155648-60-5, purity > 99.5%) was obtained from Chemlin Chemical Industry Co., Ltd (Nanjing, China), calcium chloride (CaCl_2_, CAS: 10043-52-4, purity > 99.9%) was obtained from Macklin Biochemical Co., Ltd (Shanghai, China). The organic solvents, including acetonitrile (MeCN), acetone (ace), *N*-methyl-2-pyrrolidinone (NMP), dimethyl sulfoxide (DMSO), formamid (MEF), *N*,*N*-dimethylformamide (DMF), *N*,*N*-dimethylacetamide (DMAC), *N*,*N*-diethylformamide (DEF) and *N*,*N*-diethylacetamide (DEAC) were analytical pure and were purchased from Macklin Biochemical Co., Ltd (Shanghai, China). Double distilled water was prepared and preserved in the laboratory and was used throughout the experiments.

### 2.2 Ca(Min)_2_ crystallization in various solvents

To investigate the morphology transformation in different organic–water solvents, a series of reaction crystallization experiments were designed and conducted. Firstly, Min and CaCl_2_ powders were dissolved in water to prepare 1 mM and 10 mM solutions, respectively. Secondly, 1 mL Min solution was added to each of six bottles, then gradient volumes (50 μL) with 50–250 μL of organic solvent were added and mixed uniformly. Thirdly, 100 μL CaCl_2_ solution were added to each bottle, then the bottles were kept at 298.15 K for 12 hours to obtain the crystals. Finally, the crystals were washed by double distilled water, and dried and collected for further characterizations. The same operations were applied to all nine organic solvents.

### 2.3 Characterizations

The crystal morphology of Ca(Min)_2_ was observed and captured through an inverted fluorescence microscope (TS100, Nikon, Japan) and a scanning electron microscope (Q25, FEI, USA). Powder X-ray diffraction (PXRD) was carried out on a diffractometer (X'pert PRO, PANalytical, Netherlands) using Cu-Kα radiation. The data were recorded at room temperature with the scan range being 5–90° and steps being 0.01313°.

### 2.4 Simulations

#### 2.4.1 The attachment energy (AE) model

In our simulations, the AE model was applied. It is put forward that the relative growth rate *R*_*hkl*_ of a crystal face (*h k l*) is proportional to the value of its attachment energy *E*_att_:*R*_*hkl*_ ∝ |*E*_att_|

And *E*_att_ indicates the energy released when adding a growth slice onto the crystal surface, which is calculated as:*E*_att_ = *E*_latt_ − *E*_slice_where *E*_latt_ represents the lattice energy, and *E*_slice_ is the energy of growing a slice with thickness *d*_*hkl*_.

As is known to all, most crystals are grown from solutions, thus the effect of the solvent on crystal growth plays an important role. However, the AE model does not include the adsorption of the solvent at the crystal–solvent interface, which will normally inhibit crystal growth.^25^ Herein, a modified AE model was proposed. It is known that there is energy consumption when removing adsorbed solvents from a crystal face, then the attachment energy decreases and the crystal morphology in solution is different from that in vacuum.

In this work, the modified attachment energy *E*_att_′ is computed as follows:*E*_att_′ = *E*_att_ − *E*_int_where *E*_int_ represents the interaction energy between the crystal face and the solvent layer, which is computed by this formula:*E*_int_ = *E*_tot_ − (*E*_cry_ + *E*_sol_)where *E*_tot_ is the total energy of the crystal–solvent model, *E*_cry_ is the energy of the crystal face, and *E*_sol_ is the energy of the solvent layer. Correspondingly, the relative growth rate *R*_*hkl*_′ of the crystal face and *E*_att_′ share similar proportional relation:*R*_*hkl*_′ ∝ |*E*_att_′|

#### 2.4.2 Structural models

**Fig. 1:**
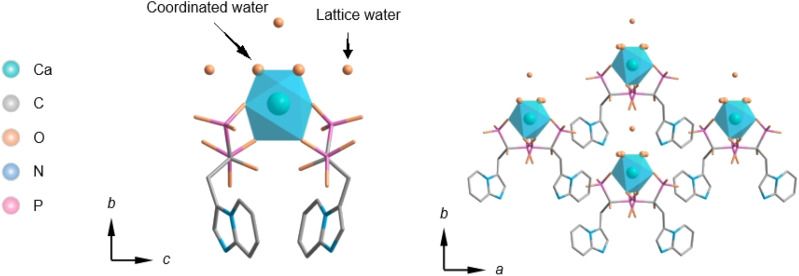
Parallel and central view of Ca(Min)_2_ crystal structure.

The crystal structure file of Ca(Min)_2_ was deposited to the Cambridge Crystallographic Data Centre (CCDC) (Deposition Number: 2172740), and the crystal was in the *C*2/*c* space group with lattice parameters *a* = 19.40 Å, *b* = 9.78 Å, *c* = 17.05 Å, *β* = 106.44° and *ρ* = 1.65 g cm^−3^, as shown in Fig. 1. Ca(Min)_2_ took a 0-D polymer structure, The Ca^2+^ centre in Ca(Min)_2_ was coordinated by two Min ions and two coordination water molecules, and there were another three lattice water molecules in the units.

Further molecular simulations were performed and completed with Materials Studio 8.0 software (Accelrys Inc., USA). The atomic charges of water molecule were appointed with *q*(O) = −0.72*e*, *q*(H) = 0.36*e* in Dreiding force field and *q*(O) = −1.22*e*, *q*(H) = 0.61*e* in Universal force field, in which case the water density by simulation is the closest to the experiment. After optimization using different forcefield/charge rules (Table S1†), Dreiding force field and Gasteiger charge calculation methods (for Ca and Min) were the most appropriate. An optimized cell with *a* = 19.27 Å, *b* = 9.74 Å, *c* = 17.05 Å, *β* = 107.99° and *ρ* = 1.69 g cm^−3^ was obtained, showing an acceptable structural deformation. The atomic charge of organic molecules were calculated by GGA/PBE functional theory and TNP basis set with electrostatic potential (ESP) fitting. The density deviation of 25 vol% organic–water solvents between experiment and simulation (1000 molecules, Dreiding force field, NPT ensemble and 500 ps) was also acceptable (<7%, Table S2†).

The construction process of the Ca(Min)_2_–organic–water interfacial model was displayed in Fig. S1.† First, the prediction of Ca(Min)_2_ crystal morphology in vacuum was conducted with the AE model and the morphological important faces were achieved. Then crystal of Ca(Min)_2_ was cleaved along these crystal face with a reasonable depth and construct superstructures at the *U* × *V* dimensions, ensuring that additional interaction calculations were avoided, see Table S3† for detailed constructions. A certain quantity of randomly distributed organic molecules were constructed at the experimental density (Table S4†) as the organic layer, and 500 randomly distributed water molecules were also constructed (1.0 g cm^−3^) as the water layer. The volume ratio of organic/water was set to 1/3, a little higher than that of the experimental method. The size of the amorphous cells was properly set as the same with the *U* × *V* of the corresponding crystal face. A vacuum layer with thickness of 100 Å was built above the solvent layer to eliminate the extra crystal–solvent interactions caused by periodic boundary conditions.

#### 2.4.3 Molecular dynamics (MD)

The Ca(Min)_2_ growth faces were constrained in MD simulations because there should be no structural relaxation of a crystal surface in the AE model. Firstly, the geometry optimization was carried out. The electrostatic interactions were calculated by the Ewald approach and the van der Waals forces were evaluated by the atom-based method with the cutoff distance of 15.5 Å. Then the MD simulations were computed at 298.15 K under the NVT ensemble and the NHL thermostat for 500 ps with a time step of 1 fs for the uniform distribution of solvent molecules, and the trajectories were collected every 500 time steps. The first 250 ps of the trajectories was for the model equilibrium and the last 125 ps of the trajectories was used for subsequently analysis.

## 3. Results and discussion

### 3.1 Intermolecular interactions

The PXRD patterns were illustrated in Fig. S2† for polymorph identification. It was obvious that patterns of crystals in all nine organic–water solvents were the same with the simulated from Ca(Min)_2_ crystal structure and there was no polymorphic transformation, so calculations based on Ca(Min)_2_ crystal structure were reliable and applicable to this study. The assumption of the AE model was on the basis of the crystallography geometry and the intermolecular interactions of the crystal, so to calculate the intermolecular interactions in the Ca(Min)_2_ crystal was important and necessary. Basically, the connection between Ca and O atoms and Hirshfeld surface analysis^50^ were taken into consideration.

Since AE model could not predict morphology of infinite networks, analysis of the Ca(Min)_2_ in terms of bonds between Ca and O atoms was necessary. As was shown in Fig. S3,† the distance between Ca and O atoms were above standard ion radius 2.21 (= 1.21 + 1.00, Table S5†), so the bonds between Ca and O were mainly ionic, thus there were no connection of Ca–O in AE model.

**Fig. 2:**
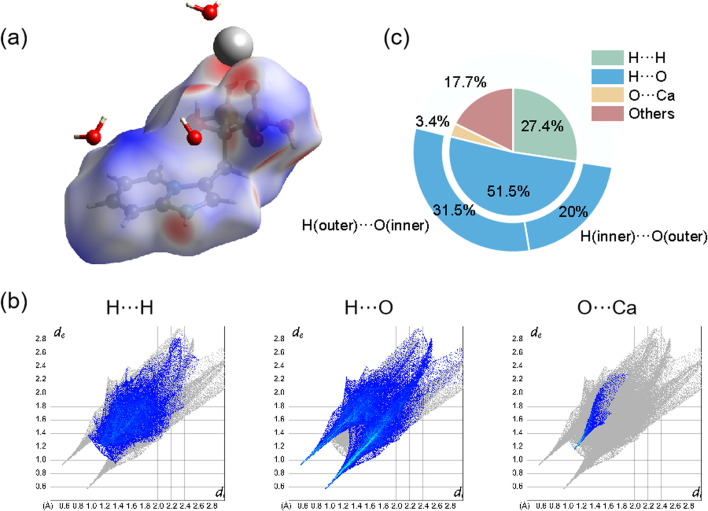
Hirshfeld surface analysis of Ca(Min)_2_. (a) 2D fingerprint plots; (b) 3D dnorm surface; (c) the relative contribution of different interactions to the Hirshfeld surface.

The intermolecular interactions were also demonstrated and calculated by Hirshfeld surface analysis. As shown in Fig. 2(a), the red spots of the surface were highlighted to represent the sites of ionic bonding and hydrogen bonding, including strong ionic bonding formation between Ca atoms and O atoms of Min and strong hydrogen bonding formation between O atoms of H_2_O and H atoms of Min or between H atoms of H_2_O and O atoms of Min. Fig. 2(b) illustrated the 2D fingerprint plot of Ca(Min)_2_, reflecting the characteristics of intermolecular interactions. It was seen that these interactions were mainly consist of H⋯H, H⋯O, O⋯Ca and others. Fig. 2(c) showed the relative contribution of different interactions to the Hirshfeld surface in the 2D fingerprint. It was obvious that the H⋯O contacts between H_2_O and Min molecules were the most frequent interactions, which comprises 51.5% area. H⋯H and O⋯Ca interactions were relatively strong in similar degrees, making up 27.4% and 3.4%, respectively. Besides, there were also other interactions (such as C⋯C) occupying 17.7% in the crystal. It was concluded that ionic bonding, hydrogen bonding and van der Waals force made most contributions, revealing that the three interactions were dominantly intermolecular contacts in the structure. Besides, detailed analysis of H⋯O interaction shows that H (inner)⋯O (outer) accounts for 20%, representing the interaction of Min as a hydrogen bond donor, and H (outer)⋯O (inner) accounts for 31.5%, representing the interaction of Min as a hydrogen bond receptor. The possible reason for the difference is the presence of crystal water and two enantiomeric Min molecules.

### 3.2 Ca(Min)_2_ crystal morphology in vacuum

**Fig. 3:**
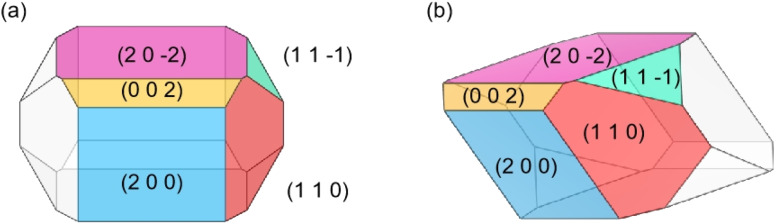
Morphology (front (a) and 45° side (b) views) of Ca(Min)_2_ predicted in vacuum.

The predicted crystal morphology of Ca(Min)_2_ in vacuum by the AE model was demonstrated in Fig. 3. Five morphologically important crystal faces named (2 0 0), (1 1 0), (0 0 2), (1 1 1̄) and (2 0 2̄) were illustrated in different colors. Table 1 listed the parameters of the morphology prediction computed by the AE model in vacuum. It showed that the (0 0 2) face owned the largest |*E*_att_| (365.24 kcal mol^−1^) and thus leading to the smallest facet area (4.54%), while the (2 0 2̄) face owned the smallest |*E*_att_| (185.29 kcal mol^−1^) and thus leading to the largest facet area (35.10%). Generally, the |*E*_att_| sequence of these five crystal faces was (0 0 2) > (1 1 1̄) > (1 1 0) > (2 0 0) > (2 0 2̄), however, the area of (1 1 0) was higher than (2 0 0).

**Table 1:** Crystal morphology parameters of Ca(Min)_2_ computed by the AE model in vacuum

(*h k l*)	Multiplicity	*d*_*hkl*_, Å	*E*_att_, kcal mol^−1^	*R*_*hkl*_	Total facet area, %
(2 0 0)	2	9.16	−250.08	1.00	20.38
(1 1 0)	4	8.60	−266.26	0.35	30.96
(0 0 2)	2	8.11	−365.24	2.96	4.54
(1 1 1̄)	4	8.10	−284.40	0.81	9.03
(2 0 2̄)	2	7.29	−185.29	0.61	35.10

The predicted morphology of Ca(Min)_2_ crystal by the AE model was enclosed by five crystal faces, being distinct from the experimental crystal morphology in organic–water solvents. Thus, it was necessary to take solvent effects on the morphology of Ca(Min)_2_ crystal into consideration.

To explore the contact of solvent molecules with Ca(Min)_2_ surface, the solvent-accessible area of different Ca(Min)_2_ crystal faces were demonstrated in Fig. S4.† The pink region on each crystal face represented the solvent-accessible surface. It was obvious that the (2 0 0) and (1 1 1̄) faces were rough with large cavities increasing the contact area, while the (1 1 0), (0 0 2) and (2 0 2̄) habit faces were relatively even. The parameter *S* was used to quantitatively evaluated the adsorption sites of solvent molecules adsorbing onto crystal faces, which could be calculated by the ratio of the solvent-accessible area (*A*_acc_) to the crystal face area (*A*_*hkl*_), as listed in Table S6.† A larger *S* value means a rougher surface and more possible adsorption sites. It was found that the *S* values of (2 0 0) and (1 1 1̄) faces have relatively large, indicating complex molecular arrangement of crystal surfaces. It implies that the adsorption of solvent molecules was favorable. For (1 1 0), (0 0 2) and (2 0 2̄) faces, the relatively low *S* values mean morphological smoothness, which is not conducive to the adsorption behavior of solvents. However, in consideration of surface structures and polar groups, it was found that other than the exposure of polar water molecules and protonated imidazolopyridine rings of all five surfaces, only (1 1 0) face exposed polar unsaturated Ca atoms. This revealed that all five faces was considered as polar surfaces, and (1 1 0) face implied to have the strongest capability of adsorbing polar solvents.

### 3.3 Ca(Min)_2_ crystal morphology in organic–water solvents

The solvent effects on the Ca(Min)_2_ morphology was investigated through molecular dynamic simulations by the modified AE model. The organic solvents selected in this study were miscible in water and could significantly accelerate crystallization. The calculation parameters of the predicted morphology were listed in Table S7.†. In each solvent system, *E*_int_ was found to be negative on all five crystal faces, suggesting that the interactions between the crystal face and the solvent layer were spontaneous. By using the (1 1 0) face as an example, different values of *E*_int_ implied that these solvents had obviously varied influences on the (1 1 0) face, due to their specific characteristic in molecular level. In terms of the total facet areas, the (0 0 2) and (1 1 1̄) faces were absent in morphology in all the solvents due to relatively higher growth rates than those of other faces. The total facet area of the (2 0 0) face in all solvents followed the sequence: DEF > ace > DMAC > DEAC > DMF > NMP > MeCN > DMSO > MEF, which revealing that the solvent effects were different on the crystal face. From the perspective of molecular structure and volume, alkylamide solvents (DMF, DMAC, DEF and DEAC) have a great influence on the crystal morphology of Ca(Min)_2_.

**Fig. 4:**
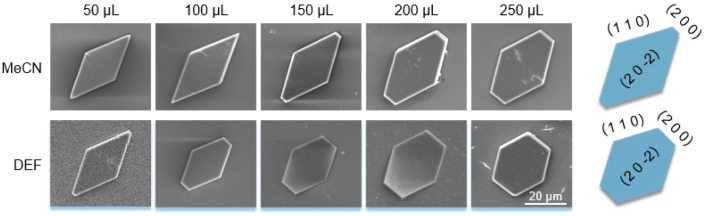
Experimental morphology of Ca(Min)_2_ after adding 50–250 μL organic solvents (left five SEM photos) and simulated morphology in 25 vol% organic–water solvent (right pictures, corresponding to a little higher than 250 μL in experiment).

**Fig. 5:**
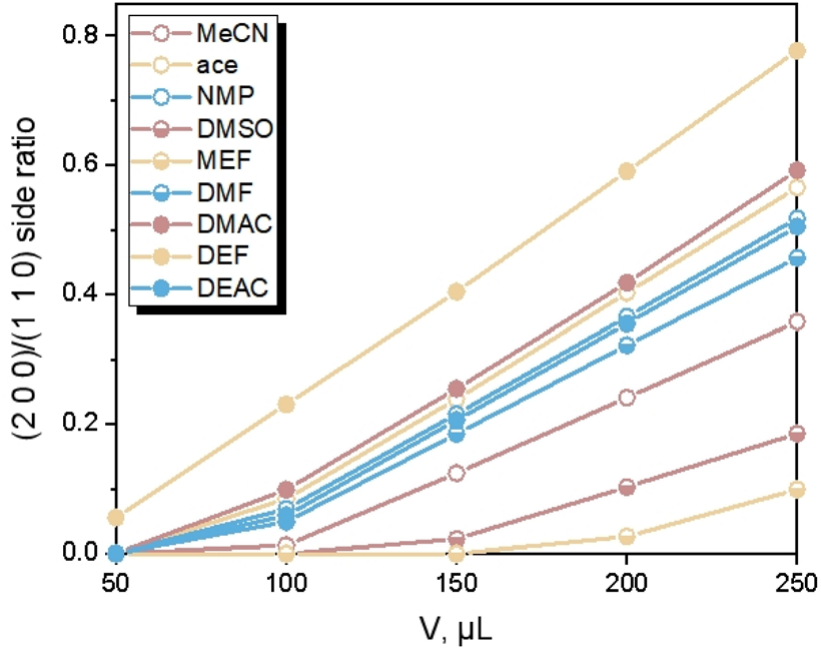
Change of (2 0 0)/(1 1 0) side ratio with volume of organic solvents.

Different from the Ca(Min)_2_ morphology prediction with the AE model (Fig. 3), the morphology prediction with the modified AE model included only three faces, *e.g.* (2 0 0), (1 1 0) and (2 0 2̄) faces, as shown in Fig. 4 (only contained the Ca(Min)_2_ morphology obtained from MeCN–H_2_O to DEF–H_2_O as examples because the trend in other organic–water solvents was similar, and the experimental and predicted morphologies in other solvents were displayed in Fig. S5†). It was also obvious that the (0 0 2) and (1 1 1̄) faces were absent in the predicted morphologies, which was consistent with the experimental appearances. These results indicated that it was reasonable to apply the modified AE model to demonstrating the solvent effects on the Ca(Min)_2_ crystal morphology. Moreover, we defined (2 0 0)/(1 1 0) side ratio as the value based on optical photos (Fig. S6†) to quantitatively evaluate the influence of different organic solvents on the crystal morphology. It could be found from Fig. 5 that the side ratio is basically linear with the volume of organic solvent. When the volume of organic solvent reached 250 μL, the crystal morphology in DEF–H_2_O had the largest side ratio of 0.78, while the morphology in MEF–H_2_O had the smallest side ratio of 0.10, and the influence order of nine organic solvents is DEF > DMAC > ace > NMP > DEAC > DMF > MeCN > DMSO > MEF, which is basically consistent with the prediction results.

### 3.4 Crystal–solvent interactions on the interface

Apparently, there were relationships between Ca(Min)_2_ morphology and solvent properties. Solvents with different properties resulted in different adsorption behaviors onto specific crystal face, thus influence the crystal–solvent interactions. In this part, the molecular arrangement of organic–water solvents on (1 1 0) face were thoroughly studied to reveal the effect of volume of solvent molecules and some other physico-chemical properties of organic solvents were also included to reveal the relationships with side ratio.

#### 3.4.1 Geometry characteristics of different surfaces

**Fig. 6:**
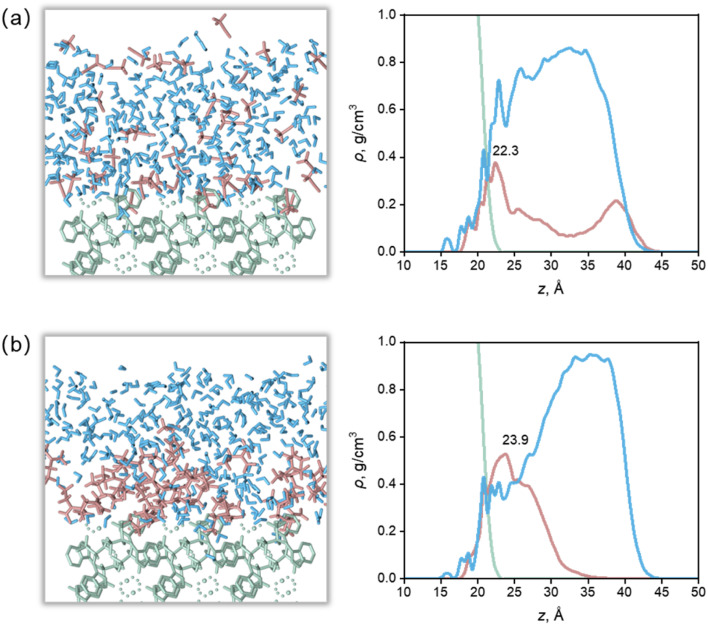
Configurations of surface–solvent interfaces (left) and mass density profile along the normal to the crystal faces (right) for the (1 1 0) face from the MD equilibrium. (a) Ca(Min)_2_–MeCN–H_2_O interfaces. (b) Ca(Min)_2_–DEF–H_2_O interfaces. Surface is in green, organic solvent is in red, and water is in blue.

The arrangement of solvent molecules on crystal faces played an important role in the crystal morphology. As mentioned before, the *E*_att_ of the (2 0 0) and (1 1 0) faces were the main reason for different effects of solvents on crystal morphology. Moreover, the increase of *E*_att_ of (1 1 0) face dominated the influence of the whole crystal morphology (defined as (2 0 0)/(1 1 0) side ratio). Hence, The equilibrium configuration of the solvent on the (1 1 0) face were applied to studying the distribution in nine solvents, which contained Ca(Min)_2_, organic and water molecules. The snapshots of the (1 1 0) crystal facet was shown in Fig. 6, using Ca(Min)_2_–MeCN–H_2_O and Ca(Min)_2_–DEF–H_2_O interfaces as examples, and the other seven solvents were shown in Fig. S7.† The arrangements of solvent molecules were different on the (1 1 0) crystal facet in two solvents, leading to a distinction in terms of the geometry characteristics and the adsorption ability. It was displayed that the solvent molecules were closely adsorbed to the (1 1 0) surface, forming an adsorption layer, indicating that all the solvents had a strong adsorption effect on the crystal surface. Most organic molecules were distributed near the surface and occupied part of the position of water molecules, indicating that organic molecules have strong interaction with the surface and are not easily affected by water molecules; Some organic molecules also diffuse to the place far away from the surface and have little interaction with the surface. Relatively, ace, NMP and alkylamide solvents (DMF, DMAC, DEF and DEAC) are closely distributed, while MeCN, DMSO and MEF are loosely distributed, and more organic molecules diffuse far from the surface, indicating that these organic solvents are more susceptible to the influence of water molecules and adsorbed from the position near the surface.

#### 3.4.2 Analysis of the mass density profiles

To further investigate the distribution of solvent molecules on crystal surface, the mass density profile was introduced, which can provide the density of solvent along the normal to the crystal surface. As shown in Fig. 6 and S7,† both the organic solvent layer and the water layer overlap with the crystal surface (<22.7 Å), indicating that some organic molecules and water molecules are embedded in the depression of the rough crystal surface and have strong adsorption with it. The highest density peaks of the nine organic solvent layers are all distributed within 20–25 Å, which is near the edge of the crystal surface (∼22.7 Å), indicating that a large number of organic molecules are adsorbed on the crystal surface. Among them, the highest density peaks of MeCN, ace and MEF were at *z* = 22.3 Å. Due to their small molecular volume, it was easy to embed into the concave position of the surface. The highest density peaks of NMP (*z* = 24.0 Å), DMSO and DMF (*z* = 23.4 Å), DMAC and DEF (*z* = 23.9 Å), DEAC (*z* = 24.4 Å) were all above 22.7 Å. Because of their large molecular volume, it was difficult to embed into the concave position of the crystal plane, thus they formed fewer short-range forces such as coordination bonding, hydrogen bonding and van der Waals force with the surface. It was worth noting that ace, DMSO and DMF have similar structures, but the positions of the highest density peaks are different, which indicates that the molecular configuration and conformation were influential.

The highest density peaks of the water layer in the nine solvents were all above 30 Å and the density value were close to the experimental, indicating that most water molecules are sterically hindered by organic molecules, and cannot form strong short-range adsorption with the crystal surface. For MeCN, DMSO and MEF, there were some organic molecules at *z* ≥35 Å, where there were almost no organic molecules in other solvents, indicating that MeCN, DMSO and MEF were more susceptible to the influence of water molecules and adsorbed from the surface. It is worth noting that the beginning of the mass density profile of the water layer is lower than that of the organic layer. This is because although the organic molecules are embedded in the surface, they could not fully fill all the depressions due to their large molecular volume, so the space between the organic molecules and the surface was filled by water molecules with smaller volume.

#### 3.4.3 Relations with organic solvent properties

**Fig. 7:**
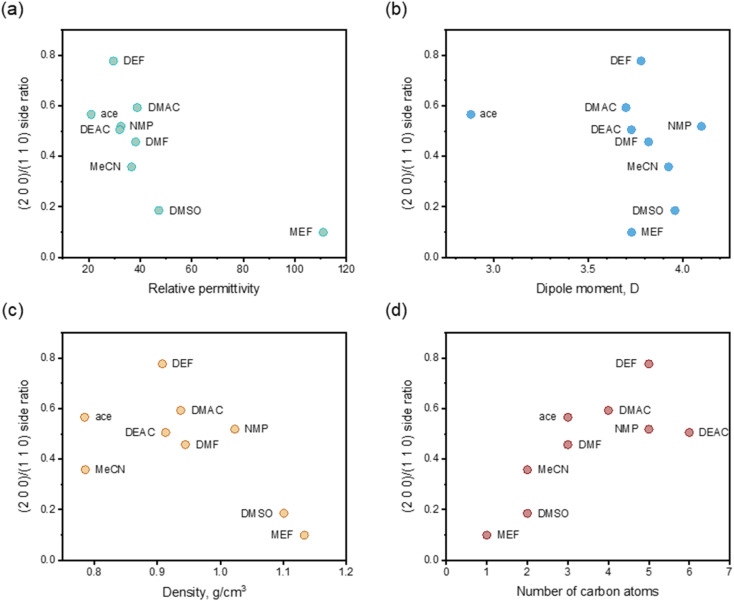
Relations between (2 0 0)/(1 1 0) side ratio and relative permittivity (a), dipole moment (b), density (c) and number of carbon atoms (d) after adding 25 vol% organic solvents.

Accordingly, the solvents considered in this work have different properties, such as relative permittivity, dipole moment, density of organic solvent and number of carbon atoms. In order to get the dominating factors or properties that determines the (2 0 0)/(1 1 0) side ratio, the relationships between the side ratio (after adding 25 vol% organic solvents) and the properties was determined (Fig. 7). It can be seen that the side ratio has a certain correlation with these four factors, and generally decreases with the increase of dielectric constant, dipole moment and density, and increases with the increase of carbon atom number. Since the dielectric constant and dipole moment represented the polarity to a great extent, and the number of carbon atoms had a positive correlation with the molecular volume, it was also reasonably considered that the side ratio decreases with the increase of solvent polarity and increases with the increase of molecular volume. The relations might be because the larger the dielectric constant and dipole moment of the solvent, the stronger the interaction force with the polar crystal surface. Similarly, the higher the density or the fewer the number of carbon atoms, the more interaction sites with the crystal surface, tending to form larger interaction energy. The larger interaction energy will lead to the higher growth rate, and the final (2 0 0)/(1 1 0) side ratio of crystal morphology appears smaller. Due to the limited selection of organic solvents, these trends needed to be further verified.

## 4. Conclusions

The organic solvent effect on the crystal morphology of Ca(Min)_2_ was systematically investigated. The predicted morphologies of Ca(Min)_2_ in different organic–water solvents by modified AE model were in well consistent with the corresponding experimental observations. The crystal–solvent interactions and adsorption behaviors were obtained using MD simulations and the results demonstrated that organic solvents would decrease the value of *E*_att_′ of all five morphological important faces and finally resulted in the disappearance of (0 0 2) and (1 1 1̄) surfaces. Furthermore, the (2 0 0)/(1 1 0) side ratios of predicted morphologies were different in solvents mainly due to different adsorption behaviors of organic and water molecules on (1 1 0) face. According to the experiment, the side ratio was linear with the volume of organic solvent and the influence order was DEF > DMAC > ace > NMP > DEAC > DMF > MeCN > DMSO > MEF. Additionally, the side ratio was approximately negatively related to the relative permittivity, dipole moment and density of organic solvent, and positively related to the number of carbon atoms in organic molecules. This work may provide an effective way of altering Ca(Min)_2_ crystal morphology for improvement of their reprocessing, filling property and biodegradability, and give a guidance in selection and addition of organic solvents to control the desirable crystal morphology.

## Author contributions

This work was planned and proceeded by discussion among Chen Zhuang, Muyuan Chai, Yuhui Zhang and Xuetao Shi. All experiments and simulations were completed by Chen Zhuang. The analysis of experimental results was assisted by Muyuan Chai and the parametric construction of simulations was advised by Yuhui Zhang. The manuscript was revised and decided by Xuetao Shi.

## Conflicts of interest

There are no conflicts to declare.

## Supplementary Material

RA-013-D2RA07130D-s001

## References

[cit1] Yi Q., Chen J., Le Y., Wang J., Xue C., Zhao H. (2013). J. Cryst. Growth.

[cit2] Liang Z., Chen J. F., Ma Y., Wang W., Han X., Xue C., Zhao H. (2014). CrystEngComm.

[cit3] Gu C. H., Young Jr V., Grant D. J. W. (2001). J. Pharm. Sci..

[cit4] Li J., Doherty M. F. (2017). Cryst. Growth Des..

[cit5] Benmessaoud I., Koutchoukali O., Bouhelassa M., Nouar A., Veesler S. (2016). J. Cryst. Growth.

[cit6] Hod I., Mastai Y., Medina D. D. (2011). CrystEngComm.

[cit7] Singh M. K., Banerjee A. (2013). Cryst. Growth Des..

[cit8] Urbelis J. H., Swift J. A. (2014). Cryst. Growth Des..

[cit9] Shim H. M., Kim H. S., Koo K. K. (2015). Cryst. Growth Des..

[cit10] Sangwal K., Zdyb A., Chocyk D., Mielniczek-Brzöska E. (1996). Cryst. Res. Technol..

[cit11] Lan Z., Calligaris G. A., De Menezes A. S., Dos Santos A. O., Lai X., Cardoso L. P., Roberts K. J. (2018). Cryst. Growth Des..

[cit12] Kuvadia Z. B., Doherty M. F. (2013). Cryst. Growth Des..

[cit13] Jennings J., Butler M. F., McLeod M., Csányi E., Ryan A. J., Mykhaylyk O. O. (2018). Cryst. Growth Des..

[cit14] Siegfried M. J., Choi K. S. (2006). J. Am. Chem. Soc..

[cit15] Song L. C., Lv S., Guo H., Cui Y. B., Zhang X., Zhang S. G., Tian Y., Yang C. H. (2022). J. Chem. Thermodyn..

[cit16] Cui P. P., Yin Q. X., Zhang S. H., Cheng X. W., Dai J. Y., Zhang Z. X., Zhou L., Xie C. (2020). J. Cryst. Growth.

[cit17] Donnay J. D. H., Harker D. (1937). Am. Mineral..

[cit18] Docherty R., Clydesdale G., Roberts K. J., Bennema P. (1991). J. Phys. D: Appl. Phys..

[cit19] Berkovitch-Yellin Z. (1985). J. Am. Chem. Soc..

[cit20] Zepeda-Ruiz L. A., Maiti A., Gee R., Gilmer G. H., Weeks B. L. (2006). J. Cryst. Growth.

[cit21] Maiti A., Gee R. H. (2009). Propellants, Explos., Pyrotech..

[cit22] Seo B., Kim S., Lee M., Kim T., Kim H. S., Lee W. B., Lee Y. W. (2018). Cryst. Growth Des..

[cit23] Shim H. M., Koo K. K. (2014). Cryst. Growth Des..

[cit24] Shim H. M., Koo K. K. (2015). Cryst. Growth Des..

[cit25] Liu Y., Niu S., Lai W., Yu T., Ma Y., Gao H., Zhao F., Ge Z. (2019). CrystEngComm.

[cit26] Li J., Jin S., Lan G., Xu Z., Wu N., Chen S., Li L. (2019). J. Cryst. Growth.

[cit27] Liu N., Li Y., Zeman S., Shu Y., Wang B., Zhou Y., Zhao Q., Wang W. (2016). CrystEngComm.

[cit28] Zhao Q., Liu N., Wang B., Wang W. (2016). RSC Adv..

[cit29] Wagle D. V., Zhao H., Baker G. A. (2014). Acc. Chem. Res..

[cit30] Niederberger M., Garnweitner G., Buha J., Polleux J., Ba J., Pinna N. (2006). J. Sol-Gel Sci. Technol..

[cit31] KOBAYASHI M., KATO H., KAKIHANA M. (2012). J. Ceram. Soc. Jpn..

[cit32] Han Y., Liu S., Han M., Bao J., Dai Z. (2009). Cryst. Growth Des..

[cit33] Liu J., Xu B., Song C., Luo H., Zou X., Han L., Yu X. (2012). CrystEngComm.

[cit34] Liu D., Kramer S. A., Huxford-Phillips R. C., Wang S., Della Rocca J., Lin W. (2012). Chem. Commun..

[cit35] Villanneau R., Racimor D., Messner-Henning E., Rousselière H., Picart S., Thouvenot R., Proust A. (2011). Inorg. Chem..

[cit36] Kunnas Hiltunen S., Haukka M., Vepsäläinen J., Ahlgrén M. (2009). Eur. J. Inorg. Chem..

[cit37] Kontturi M., Kunnas-Hiltunen S., Vepsäläinen J. J., Ahlgrén M. (2006). Solid State Sci..

[cit38] Kontturi M., Peräniemi S., Vepsäläinen J. J., Ahlgrén M. (2005). Polyhedron.

[cit39] Schmidt C., Ulrich J. (2012). J. Cryst. Growth.

[cit40] Wu C., Xie Y. (2009). Chem. Commun..

[cit41] Yang H. G., Sun C. H., Qiao S. Z., Zou J., Liu G., Smith S. C., Cheng H. M., Lu G. Q. (2008). Nature.

[cit42] Variankaval N., Cote A. S., Doherty M. F. (2008). AIChE J..

[cit43] Winn D., Doherty M. F. (2000). AIChE J..

[cit44] Yin J., Zhou J., Suh J., Qiu Y., Wei D., Shen Y. (2008). Cryst. Res. Technol..

[cit45] Alvarez E., Marquez A. G., Devic T., Steunou N., Serre C., Bonhomme C., Gervais C., Izquierdo-Barba I., Vallet-Regi M., Laurencin D., Mauri F., Horcajada P. (2013). CrystEngComm.

[cit46] Boanini E., Torricelli P., Gazzano M., Fini M., Bigi A. (2013). Adv. Mater..

[cit47] Li W., Xin X., Jing S., Zhang X., Chen K., Chen D., Hu H. (2017). J. Mater. Chem. B.

[cit48] Matsumoto T., Endo I. (2020). Bone.

[cit49] Liu Q., Chen D., Ye Z., Jin Z., Ma T., Huang X. (2020). Medicine (Baltim.).

[cit50] Spackman M. A., Jayatilaka D. (2009). CrystEngComm.

