# Supplementary material for: Effect of organic solvents on calcium minodronate crystal morphology in aqueous solution: an experimental and theoretical study
Source: RSC Adv. 2023 Jan 18;13(4):2727–35. doi: 10.1039/d2ra07130d (PMC9846948; doi:10.1039/d2ra07130d)
Supplement: RA-013-D2RA07130D-s001 [file RA-013-D2RA07130D-s001.pdf]

Electronic Supplementary Material (ESI) for RSC Advances.
This journal is © The Royal Society of Chemistry 2023

Electronic supplementary information

Effect of organic solvents on calcium minodronate crystal morphology in aqueous solution：An experimental and theoretical study†

Chen Zhuang[a], Muyuan Chai[a], Yuhui Zhang*[b], Xuetao Shi*[acdef]

[a]*School of Materials Science and Engineering, South China University of Technology, Guangzhou 510640, China.*

[b]*School of Materials Science and Engineering, Xiamen University of Technology, Xiamen 361024, China. E-mail: joanmolin@foxmail.com*

[c]*National Engineering Research Center for Tissue Restoration and Reconstruction, South China University of Technology, Guangzhou 510006, China. E-mail: shxt@scut.edu.cn*

[d]*Key Laboratory of Biomedical Engineering of Guangdong Province, South China University of Technology, Guangzhou 510006, China*

[e]*Key Laboratory of Biomedical Materials and Engineering of the Ministry of Education, South China University of Technology, Guangzhou 510006, China*

[f]*Guangzhou Regenerative Medicine and Health Guangdong Laboratory, 510005, Guangzhou, China*

†Electronic supplementary information (ESI) available

Table S1 Change of cell parameters and density of $Ca(Min)_2$ after optimization using different force field/charge rules

Force field/Charge	a, Å	b, Å	c, Å	α, °	β, °	γ, °	ρ, g/cm^3
Experimental	19.40	9.78	17.05	90.00	106.44	90.00	1.65
COMPASS/Forcefield assigned	22.25	9.62	14.19	90.00	104.92	90.00	1.75
COMPASS/QEq	18.83	9.88	17.93	90.00	110.79	90.00	1.64
COMPASS/Gasteiger	18.58	10.09	17.59	90.00	112.84	90.00	1.69
COMPASS II/Forcefield assigned	19.69	8.74	20.44	90.00	121.79	90.00	1.72
COMPASS II/QEq	21.78	10.13	15.33	90.00	110.41	90.00	1.62
COMPASS II/Gasteiger	17.91	9.67	18.60	90.00	109.09	90.00	1.69
Dreiding/QEq	20.55	10.02	16.27	90.00	107.31	90.00	1.60
Dreiding/Gasteiger	19.27	9.74	17.05	90.00	107.99	90.00	1.69
Universal/QEq	16.08	11.46	18.95	90.00	107.29	90.00	1.54
Universal/Gasteiger	17.31	11.39	17.81	90.00	100.71	90.00	1.49
cvff/Forcefield assigned	19.45	10.66	16.48	90.00	111.08	90.00	1.61
cvff/QEq	20.79	11.02	15.55	90.00	107.75	90.00	1.51
cvff/Gasteiger	18.32	10.67	17.69	90.00	107.98	90.00	1.56
pcff/Forcefield assigned	24.39	9.57	13.06	90.00	107.63	90.00	1.77
pcff/QEq	19.21	10.10	16.73	90.00	108.13	90.00	1.66
pcff/Gasteiger	18.81	11.41	14.29	90.00	95.05	90.00	1.68

Table S2 Theoretical, simulated density (g/cm^3) and their deviation of 25 v.% organic-water solvents

Solvent	MeCN	ace	NMP	DMSO	MEF	DMF	DMAC	DEF	DEAC
Theoretical	0.9451	0.9439	1.0035	1.0230	1.0320	0.9839	0.9821	0.9758	0.9773
Simulated	0.9003	0.9018	0.9388	0.9603	1.0059	0.9169	0.9184	0.9181	0.9225
Deviation, %	-4.74	-4.46	-6.45	-6.14	-2.53	-6.81	-6.48	-5.92	-5.61

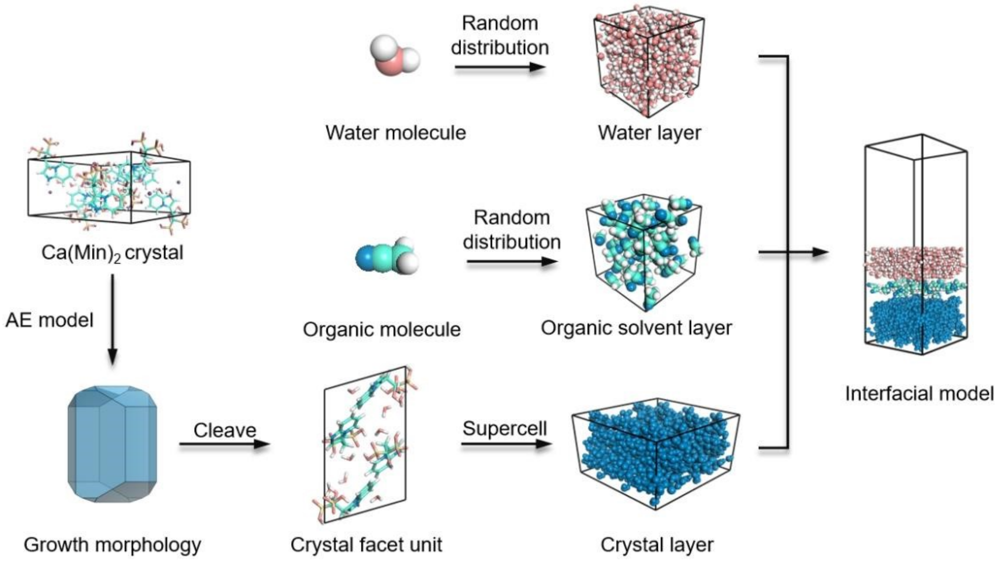

Fig. S1 Schematic diagram of the construction of the Ca(Min)$_2$-organic-water interfacial model.

Table S3 Details of the model dimensions.

(*h k l*)	d_{hkl}	U	V
(2 0 0)	×2	×4	×2
(1 1 0)	×2	×2	×3
(0 0 2)	×2	×3	×4
(1 1 -1)	×2	×2	×3
(2 0 -2)	×3	×4	×2

Table S4 Density of solvents and number of organic molecules ($V_{organic}$: V_{water} = 1: 3, 500 water molecules)

Temperature, ℃	17	19	20	25	Number of molecules
MeCN	—	—	0.7857	—	58
ace	—	—	—	0.7845	41
NMP	—	—	—	1.0230	31
DMSO	—	—	—	1.1010	42
MEF	—	—	1.1334	—	76
DMF	—	—	—	0.9445	39
DMAC	—	—	—	0.9372	32
DEF	—	0.9080	—	—	27
DEAC	0.9130	—	—	—	24
H_2O	0.9987778	0.9984079	0.9982067	0.997047	—

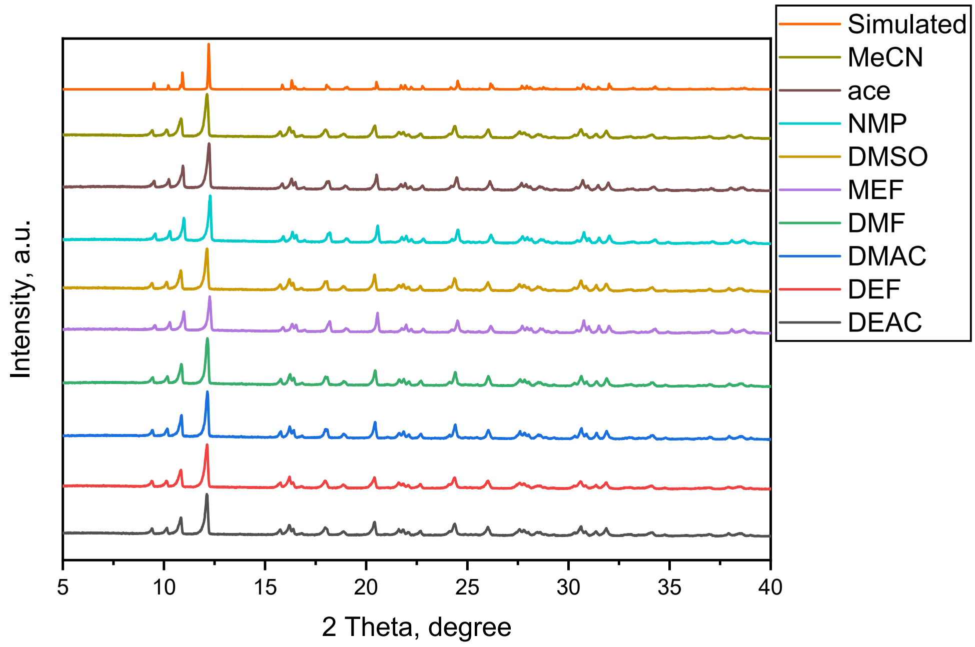

Fig. S2 PXRD patterns simulated from $Ca(Min)_2$ crystal structure and of $Ca(Min)_2$ crystals obtained from nine organic-water solvents (added 250 μL organic solvent).

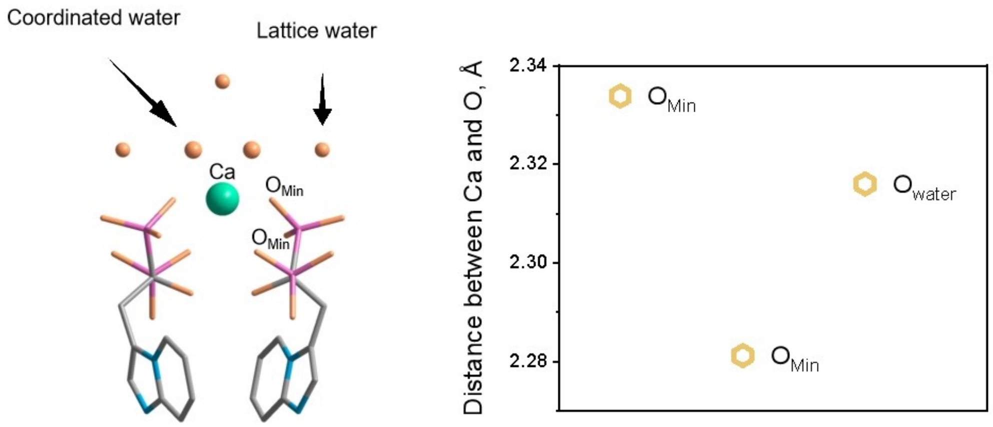

Fig. S3 Coordination distances between Ca and O in the $Ca(Min)_2$ crystal.

Table S5 Standard ion radius (according to CRC handbook of chemistry and physics 97th).

Ion	Coordination number	R_i, Å
O^{-2}	2	1.21
Ca^{+2}	6	1.00

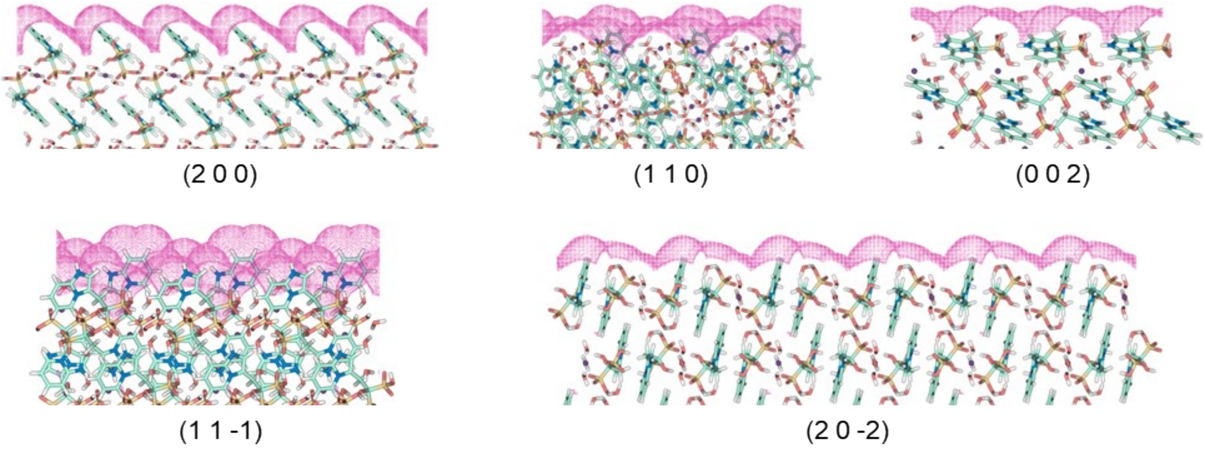

Fig. S4 Solvent accessible surfaces of $Ca(Min)_2$.

Table S6 Surface roughness (defined as $S = A_{acc} / A_{hkl}$, where A_{acc} is the solvent-accessible area of the crystal face and A_{hkl} is the area of the crystal face (h k l) both in the unit cell)

(h k l)	A_{acc}, Å^2	A_{hkl}, Å^2	S
(2 0 0)	296.61	166.10	1.79
(1 1 0)	262.43	176.93	1.48
(0 0 2)	120.96	93.84	1.29
(1 1 -1)	343.05	187.87	1.83
(2 0 -2)	289.73	208.69	1.39

Table S7 Energy (kcal/mol) and occupancy (%) of surfaces in solvents

Solvent	(h k l)	E_{tot}	E_{cry}	E_{sol}	E_{int}	E'_{att}	R'_{hkl}	Total facet area
MeCN-H_2O	(2 0 0)	-4159.28	-3142.12	-883.50	-133.66	-116.42	1.00	4.65
	(1 1 0)	-4397.69	-3015.56	-1179.73	-202.40	-63.86	0.55	58.81
	(0 0 2)	-2225.29	-1542.02	-587.90	-95.37	-269.87	2.32	—
	(1 1 -1)	-4455.16	-3102.72	-1181.40	-171.04	-113.35	0.97	—
	(2 0 -2)	-4168.41	-3164.71	-877.21	-126.48	-58.81	0.51	36.54
ace-H_2O	(2 0 0)	-4450.01	-3142.12	-1172.32	-135.57	-114.51	1.00	10.48
	(1 1 0)	-4779.52	-3015.56	-1576.75	-187.21	-79.05	0.69	47.46
	(0 0 2)	-2408.21	-1542.02	-786.85	-79.34	-285.89	2.50	—
	(1 1 -1)	-4826.53	-3102.72	-1578.81	-145.00	-139.39	1.22	—
	(2 0 -2)	-4458.97	-3164.71	-1167.84	-126.41	-58.88	0.51	42.06
NMP-H_2O	(2 0 0)	-3678.12	-3142.12	-407.98	-128.02	-122.07	1.00	6.44
	(1 1 0)	-3753.85	-3015.56	-543.33	-194.95	-71.30	0.58	56.13
	(0 0 2)	-1893.70	-1542.02	-277.80	-73.88	-291.36	2.39	—
	(1 1 -1)	-3802.99	-3102.72	-566.36	-133.91	-150.49	1.23	—
	(2 0 -2)	-3693.82	-3164.71	-407.78	-121.33	-63.96	0.52	37.43
DMSO-H_2O	(2 0 0)	-4331.33	-3142.12	-1049.75	-139.47	-110.62	1.00	3.73
	(1 1 0)	-4656.59	-3015.56	-1433.09	-207.95	-58.31	0.53	64.66
	(0 0 2)	-2325.53	-1542.02	-705.01	-78.50	-286.74	2.59	—
	(1 1 -1)	-4664.17	-3102.72	-1430.31	-131.13	-153.26	1.39	—
	(2 0 -2)	-4332.37	-3164.71	-1048.55	-119.10	-66.19	0.60	31.60
MEF-H_2O	(2 0 0)	-4533.96	-3142.12	-1240.19	-151.64	-98.44	1.00	1.83
	(1 1 0)	-4892.62	-3015.56	-1659.71	-217.36	-48.90	0.50	67.05
	(0 0 2)	-2463.56	-1542.02	-827.59	-93.95	-271.28	2.76	—
	(1 1 -1)	-4951.58	-3102.72	-1662.01	-186.85	-97.55	0.99	—
	(2 0 -2)	-4532.97	-3164.71	-1238.56	-129.70	-55.59	0.56	31.11
DMF-H_2O	(2 0 0)	-3740.99	-3142.12	-470.70	-128.17	-121.92	1.00	6.75
	(1 1 0)	-3835.80	-3015.56	-626.19	-194.05	-72.21	0.59	54.96
	(0 0 2)	-1938.27	-1542.02	-315.56	-80.70	-284.54	2.33	—
	(1 1 -1)	-3893.77	-3102.72	-645.94	-145.11	-139.28	1.14	—
	(2 0 -2)	-3751.98	-3164.71	-464.54	-122.73	-62.56	0.51	38.29
DMAC-H_2O	(2 0 0)	-3730.61	-3142.12	-465.03	-123.46	-126.62	1.00	7.77
	(1 1 0)	-3830.33	-3015.56	-626.10	-188.68	-77.58	0.61	54.25
	(0 0 2)	-1933.41	-1542.02	-312.73	-78.67	-286.57	2.26	—
	(1 1 -1)	-3879.20	-3102.72	-634.74	-141.74	-142.66	1.13	—
	(2 0 -2)	-3743.05	-3164.71	-461.41	-116.93	-68.36	0.54	37.98
DEF-H_2O	(2 0 0)	-3845.88	-3142.12	-570.51	-133.25	-116.83	1.00	12.55
	(1 1 0)	-3967.67	-3015.56	-771.62	-180.50	-85.76	0.73	47.42
	(0 0 2)	-2002.48	-1542.02	-389.28	-71.19	-294.05	2.52	—
	(1 1 -1)	-3917.90	-3102.72	-687.37	-127.81	-156.59	1.34	—
	(2 0 -2)	-3856.04	-3164.71	-575.15	-116.19	-69.10	0.59	40.03
DEAC-H_2O	(2 0 0)	-4124.79	-3142.12	-854.78	-127.89	-122.20	1.00	7.03
	(1 1 0)	-4344.75	-3015.56	-1135.52	-193.68	-72.58	0.59	56.45
	(0 0 2)	-2187.65	-1542.02	-575.52	-70.12	-295.12	2.42	—
	(1 1 -1)	-4407.78	-3102.72	-1144.09	-160.97	-123.42	1.01	—
	(2 0 -2)	-4133.99	-3164.71	-851.86	-117.42	-67.87	0.56	36.51

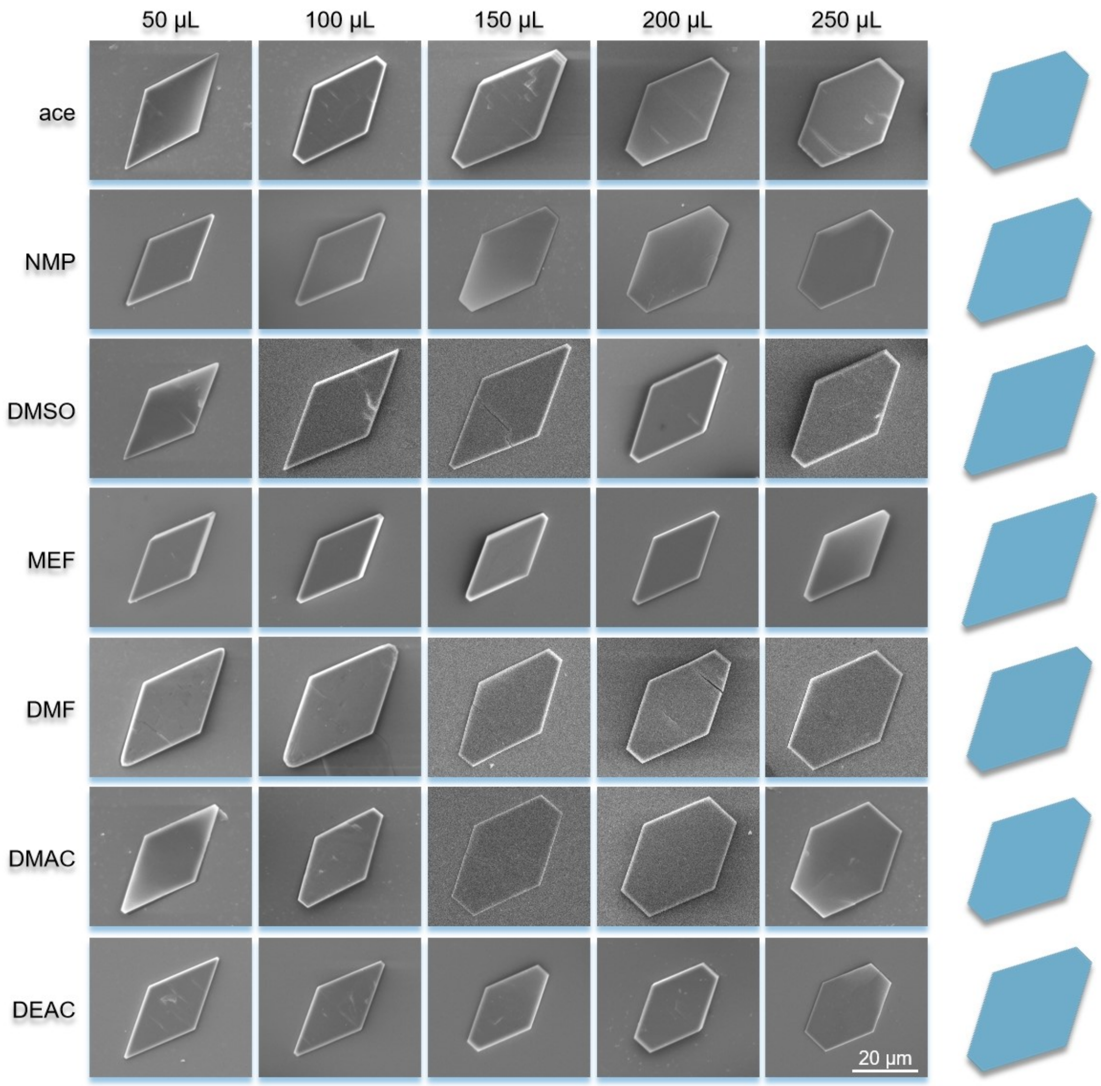

Fig. S5 Experimental morphology of $Ca(Min)_2$ after adding 50 ~ 250 μL organic solvents (left five SEM photos) and simulated morphology in 25 v.% organic-water solvent (right pictures, corresponding to a little higher than 250 μL in experiment).

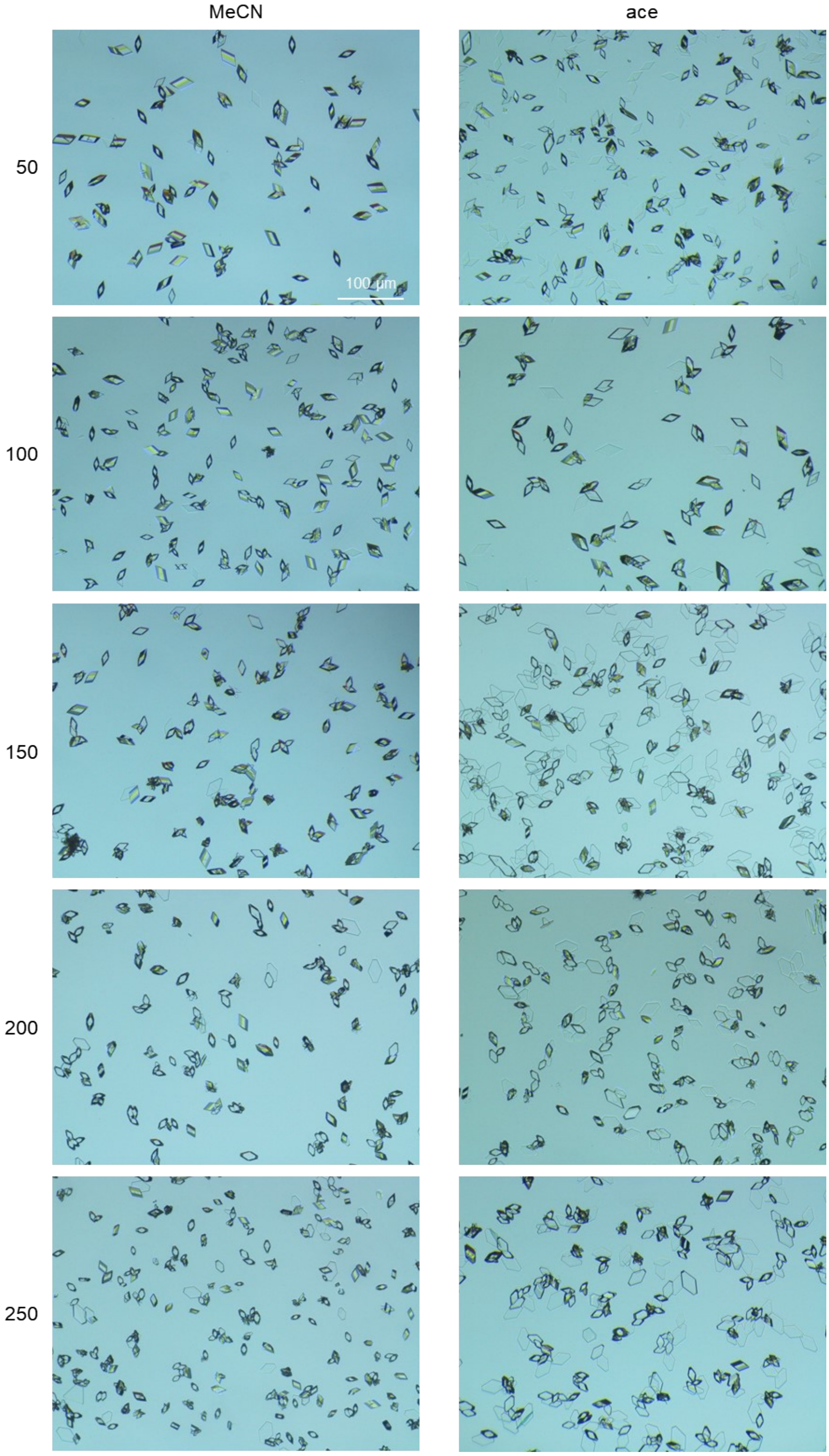

Fig. S6 Morphology of $Ca(Min)_2$ after adding 50 ~ 250 μL organic solvents

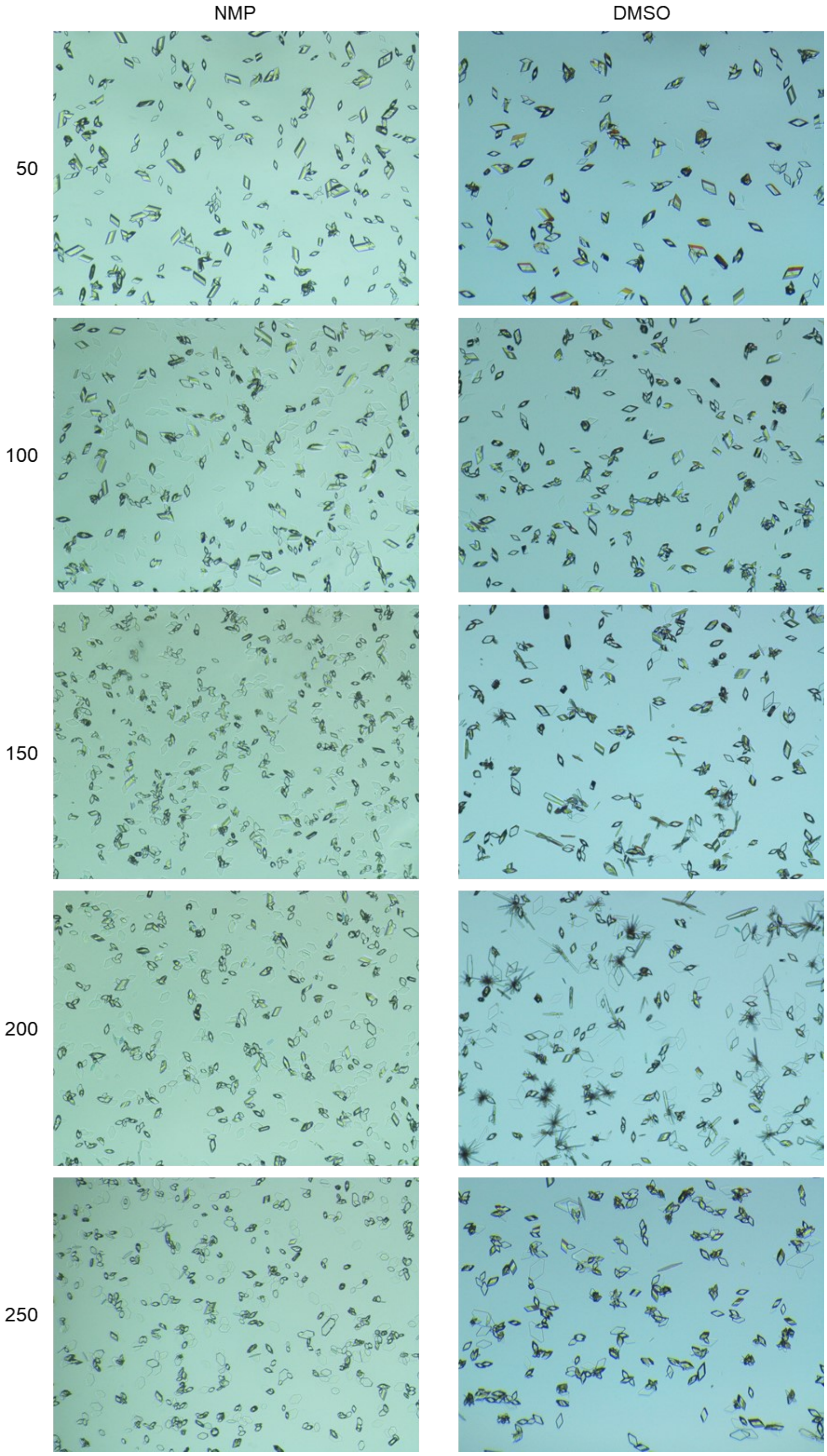

Fig. S6 Morphology of $Ca(Min)_2$ after adding 50 ~ 250 μL organic solvents (continued)

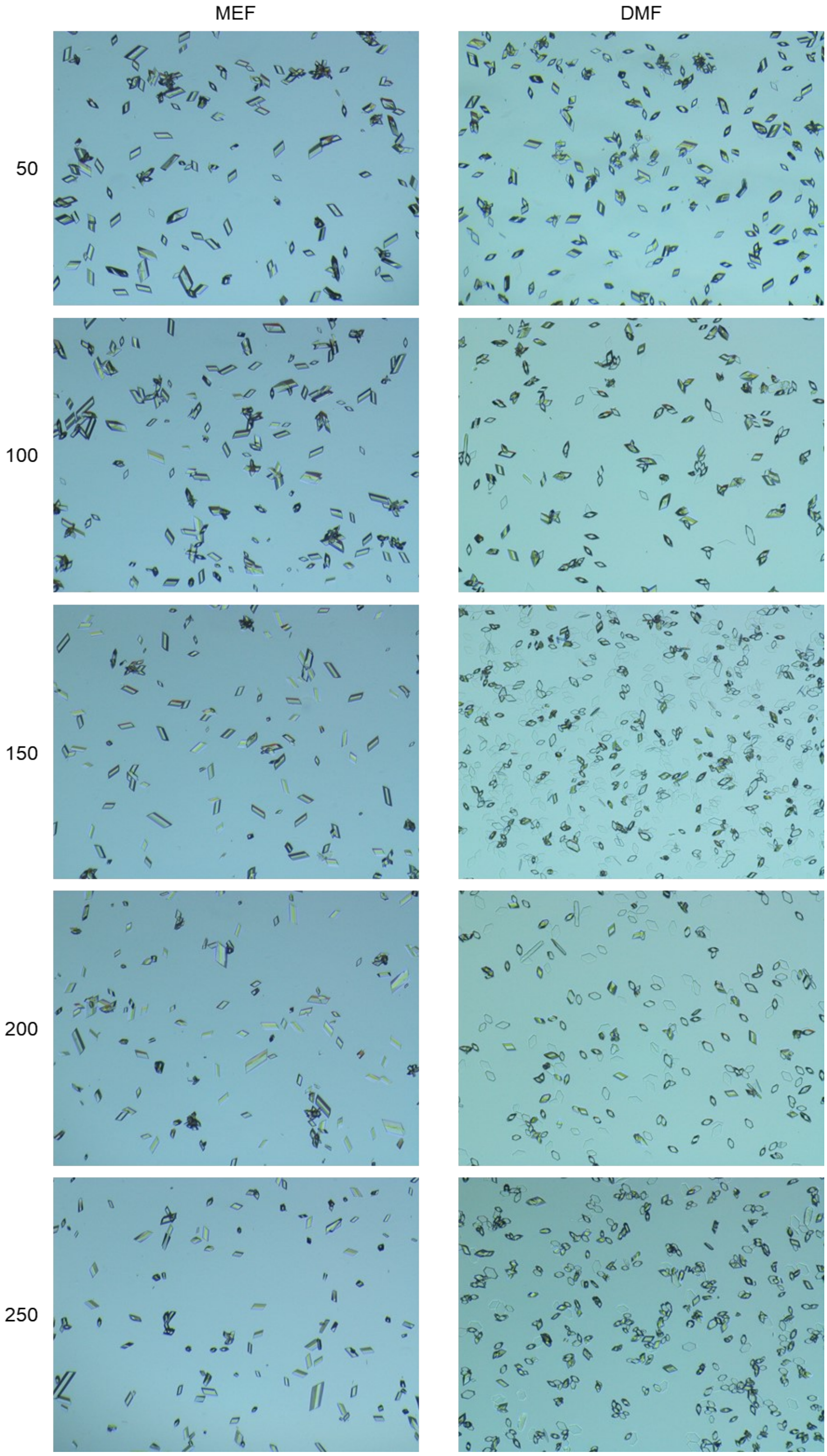

Fig. S6 Morphology of $Ca(Min)_2$ after adding 50 ~ 250 μL organic solvents (continued)

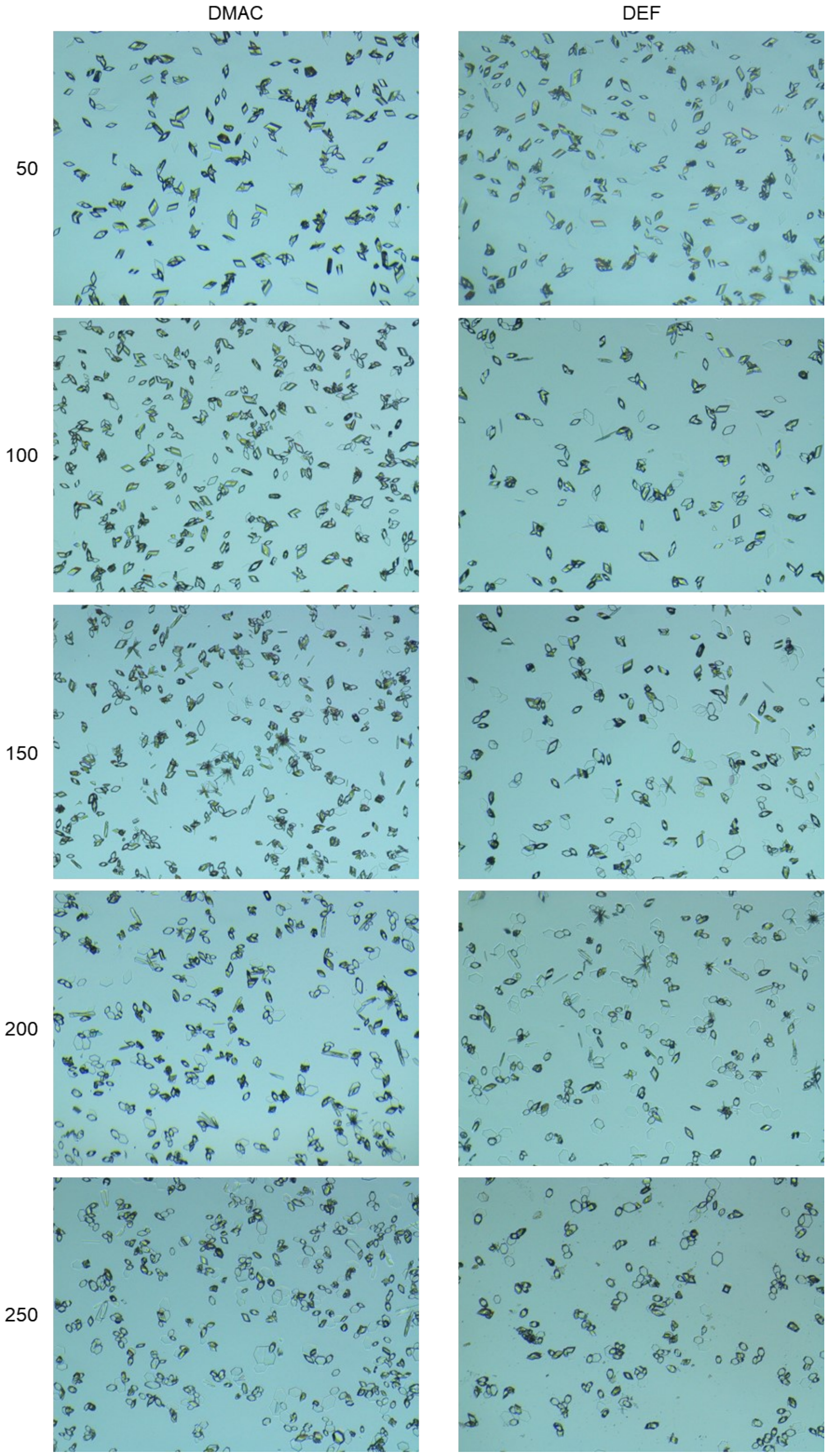

Fig. S6 Morphology of $Ca(Min)_2$ after adding 50 ~ 250 μL organic solvents (continued)

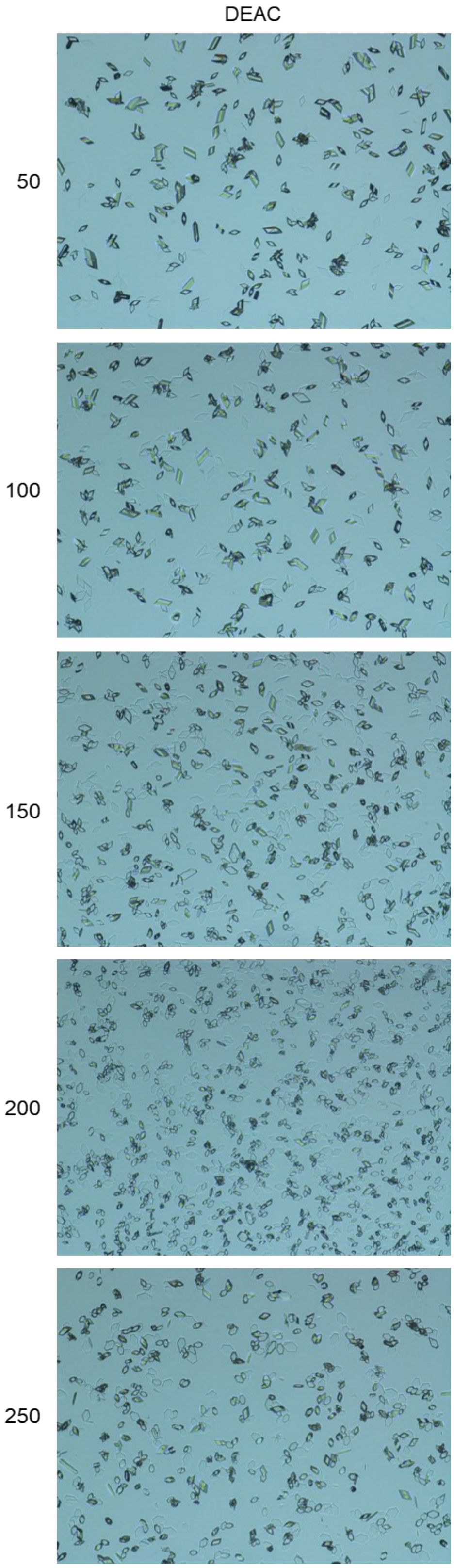

Fig. S6 Morphology of $Ca(Min)_2$ after adding 50 ~ 250 μL organic solvents (continued)

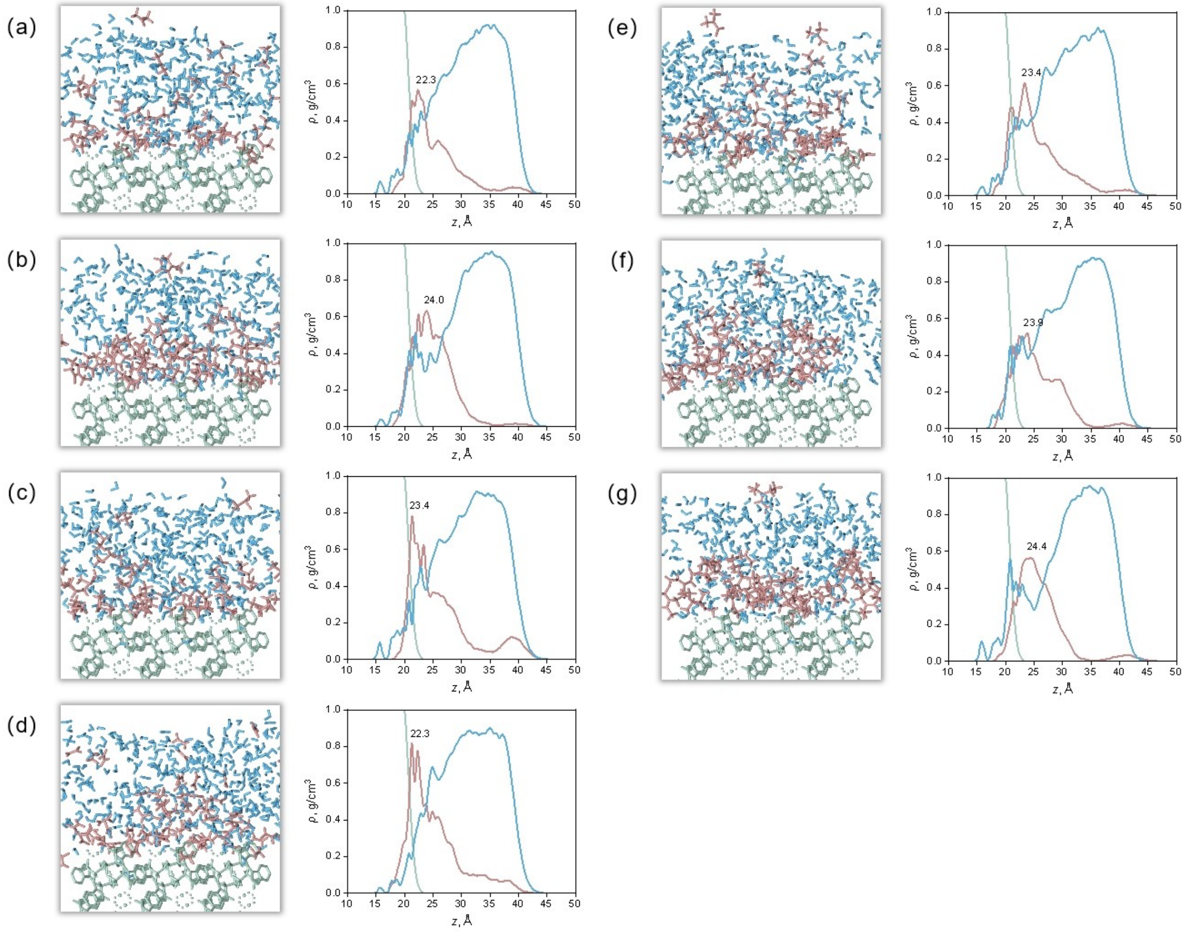

Fig. S7 Configurations of surface-solvent interfaces (left) and mass density profile along the normal to the crystal faces (right) for the (1 1 0) face from the MD equilibrium. (a) $Ca(Min)_2$-ace-H_2O interfaces. (b) $Ca(Min)_2$-NMP-H_2O interfaces. (c) $Ca(Min)_2$-DMSO-H_2O interfaces. (d) $Ca(Min)_2$-MEF-H_2O interfaces. (e) $Ca(Min)_2$-DMF-H_2O interfaces. (f) $Ca(Min)_2$-DMAC-H_2O interfaces. (g) $Ca(Min)_2$-DEAC-H_2O interfaces.Surface is in green, organic solvent is in red, and water is in blue.
